# Does happiness itself directly affect mortality? The prospective UK Million Women Study

**DOI:** 10.1016/S0140-6736(15)01087-9

**Published:** 2016-02-27

**Authors:** Bette Liu, Sarah Floud, Kirstin Pirie, Jane Green, Richard Peto, Valerie Beral

**Affiliations:** a Faculty of Medicine, University of New South Wales, Sydney, NSW, Australia; b Cancer Epidemiology Unit, Nuffield Department of Population Health, University of Oxford, Oxford, UK; c Clinical Trial Service Unit and Epidemiological Studies Unit, Nuffield Department of Population Health, University of Oxford, Oxford, UK

## Abstract

**Background:**

Poor health can cause unhappiness and poor health increases mortality. Previous reports of reduced mortality associated with happiness could be due to the increased mortality of people who are unhappy because of their poor health. Also, unhappiness might be associated with lifestyle factors that can affect mortality. We aimed to establish whether, after allowing for the poor health and lifestyle of people who are unhappy, any robust evidence remains that happiness or related subjective measures of wellbeing directly reduce mortality.

**Methods:**

The Million Women Study is a prospective study of UK women recruited between 1996 and 2001 and followed electronically for cause-specific mortality. 3 years after recruitment, the baseline questionnaire for the present report asked women to self-rate their health, happiness, stress, feelings of control, and whether they felt relaxed. The main analyses were of mortality before Jan 1, 2012, from all causes, from ischaemic heart disease, and from cancer in women who did not have heart disease, stroke, chronic obstructive lung disease, or cancer at the time they answered this baseline questionnaire. We used Cox regression, adjusted for baseline self-rated health and lifestyle factors, to calculate mortality rate ratios (RRs) comparing mortality in women who reported being unhappy (ie, happy sometimes, rarely, or never) with those who reported being happy most of the time.

**Findings:**

Of 719 671 women in the main analyses (median age 59 years [IQR 55–63]), 39% (282 619) reported being happy most of the time, 44% (315 874) usually happy, and 17% (121 178) unhappy. During 10 years (SD 2) follow-up, 4% (31 531) of participants died. Self-rated poor health at baseline was strongly associated with unhappiness. But after adjustment for self-rated health, treatment for hypertension, diabetes, asthma, arthritis, depression, or anxiety, and several sociodemographic and lifestyle factors (including smoking, deprivation, and body-mass index), unhappiness was not associated with mortality from all causes (adjusted RR for unhappy *vs* happy most of the time 0·98, 95% CI 0·94–1·01), from ischaemic heart disease (0·97, 0·87–1·10), or from cancer (0·98, 0·93–1·02). Findings were similarly null for related measures such as stress or lack of control.

**Interpretation:**

In middle-aged women, poor health can cause unhappiness. After allowing for this association and adjusting for potential confounders, happiness and related measures of wellbeing do not appear to have any direct effect on mortality.

**Funding:**

UK Medical Research Council, Cancer Research UK.

## Introduction

Happiness and related measures of wellbeing are reportedly associated with reduced mortality, particularly from heart disease.^1^, ^2^, ^3^, ^4^ Postulated mechanisms to account for this association include the possibility that happiness might itself cause biological changes, such as in serum cortisol concentration or immune function, that could in turn affect mortality.^2^, ^3^ However, serious challenges exist in interpreting the association between happiness and reduced mortality as evidence for a protective biological mechanism for happiness. Unhappiness might, for example, be associated with lifestyle factors that can cause disease,^3^ such as smoking, high alcohol consumption, obesity, or physical inactivity. Perhaps more important is reverse causality whereby poor health, which is known to be associated with an increase in mortality, can also cause unhappiness. This results in a non-causal association between unhappiness and increased mortality—or, equivalently, between happiness and reduced mortality. Our aim was to establish whether, after appropriate allowance for reverse causality and for confounding by lifestyle and sociodemographic factors, any robust evidence remains that happiness itself, or related subjective measures of wellbeing such as being in control, relaxed, or not unduly stressed, are independently associated with reduced mortality.

## Methods

### Study design and participants

From May 1, 1996, to Dec 31, 2001, the Million Women Study recruited 1·3 million women aged 50–69 years through the national Breast Screening Programmes of England and Scotland, and has continued to follow them up by electronic record linkage, recording the causes of any deaths.^5^ At recruitment, and every 3–5 years subsequently, women were posted a questionnaire asking about sociodemographic factors, lifestyle, and health.

Ethics approval was from the Anglia and Oxford multicentre research ethics committee. Access to hospital admission data was approved by the Information Centre for Health and Social Care (England) and the Information Services Division (Scotland). All study participants provided written consent.

### Procedures

At baseline 3 years after recruitment, women were asked: “How often do you feel happy?” Possible responses were “most of the time”, “usually”, “sometimes”, or “rarely/never”. They were also asked about related subjective measures of wellbeing including how often they felt in control, relaxed, and stressed. In addition, women were asked whether they had had various common health disorders and to self-rate their current health as “excellent”, “good”, “fair”, or “poor”. In the questionnaire, self-rated health came before happiness and related measures. We used data from this 3 year survey as baseline for our investigation of any associations of unhappiness (or related factors) with cause-specific mortality, and our analyses are restricted to the women who answered this question on happiness. A random sample of women were re-sent the same questionnaire about 1 year after the first one to assess the repeatability of responses.^6^

All participants in the Million Women Study are routinely followed for death (or emigration), cancer registration, and hospital admission through electronic linkage to centrally held National Health Service (NHS) records, using a combination of name, date of birth, and NHS number. Underlying causes of death, cancers, and hospital admissions are coded according to WHO Tenth International Classification of Diseases (ICD-10). Follow-up time was from the date when the baseline questionnaire on happiness was answered to whichever was first of Jan 1, 2012, or date of death or emigration.

### Outcomes and exposures

Our outcomes were mortality from all causes, from ischaemic heart disease (ICD-10 I20-I25), and from cancer (ICD-10 C00-C97). We classified women into three categories: unhappy (ie, sometimes, rarely, or never happy), usually happy, or happy most of the time. Because the latter two categories are similar, for some analyses we combined them into one category, called generally happy. Associations of mortality with other subjective measures of wellbeing (being in control, relaxed, and stressed) were also examined.

### Statistical analysis

For analyses examining which baseline factors were associated with happiness we used logistic regression (adjusted for various factors) to compare individuals who were unhappy with those who were generally happy (two-way classification). For the analyses of the association between unhappiness and mortality we used the three-way classification (with those happy most of the time as the reference group), but for clarity in the text, we report only the mortality rate ratios (RR) for unhappy versus happy most of the time. For analyses of all-cause mortality, ischaemic heart disease mortality, and cancer mortality, we used Cox proportional hazards models. We did sensitivity analyses to exclude the first 5 years of follow-up. We repeated such analyses for related measures of subjective wellbeing: being in control, being relaxed, and being stressed.

To limit reverse causality, the main mortality analyses excluded women who had already had certain illnesses (heart disease, stroke, lung disease, or cancer, as done previously^7^); additional analyses assessed the effects of these exclusions.

RRs of death were first adjusted only for age, and then additionally adjusted for various combinations of self-rated health and sociodemographic and lifestyle characteristics. These characteristics were region of residence at recruitment (Scotland and the nine cancer registration regions covering England at that time); area deprivation (quintiles, based on the Townsend Index, a score incorporating census area data for employment, car ownership, home ownership, and household overcrowding^8^); educational achievement (college [after age 18 years], A-level qualifications [usually at age 18 years], O-level qualifications [usually at age 16 years], none of these); whether living with a partner (yes, no), parity (0, 1, ≥2), body-mass index (<25 kg/m^2^, 25 to <30 kg/m^2^, ≥30 kg/m^2^); strenuous exercise (0, <3 h per week, ≥3 h per week); smoking (never, past, current <15 cigarettes per day, current ≥15 cigarettes per day); alcohol consumption (0, <7 drinks per week, ≥7 drinks per week); hours of sleep (<7 h, 7 h, 8 h, ≥9 h); and participation in religious groups (yes, no) or other group activities (yes, no). All adjustment variables were from the baseline survey (ie, at the same time that happiness and related measures were recorded), except region, deprivation, education, and parity, which were recorded at recruitment, about 3 years earlier.

We used conventional 95% CIs or 99% CIs, except in figures that compared more than two groups. For these comparisons, the variance of the log risk was estimated for each group (including the reference group).^9^ We used these group-specific variances to calculate group-specific CIs, allowing valid comparisons between any two or more groups, whether or not one of them was designated as the reference group. Analyses were done with STATA version 13.1.

### Role of the funding source

The funders of the study had no role in study design, data collection, data analysis, data interpretation, or writing of the report. The corresponding author had full access to all the data in the study and had final responsibility for the decision to submit for publication.

## Results

At baseline, a total of 845 440 women (median age 59 years; IQR 55–63) responded to the question about how often they felt happy. Replies were: 39% (329 326 women) happy most of the time, 44% (369 738) usually happy, 16% (138 678) sometimes happy, and 1% (7698) rarely or never happy. In all analyses we combined women who reported being happy sometimes, rarely, or never, and describe them as unhappy.

Among 10 143 women who completed the same happiness question twice, about 1 year apart, there was reasonably good reproducibility between the categorised responses (weighted κ for agreement 0·62). The two extreme categories had little crossover. Of 4003 women who reported being happy most of the time at baseline, only 2% (85) reported being unhappy 1 year later; conversely, of 1763 women who reported being unhappy at baseline, only 5% (81) reported being happy most of the time 1 year later.

**Figure 1:**
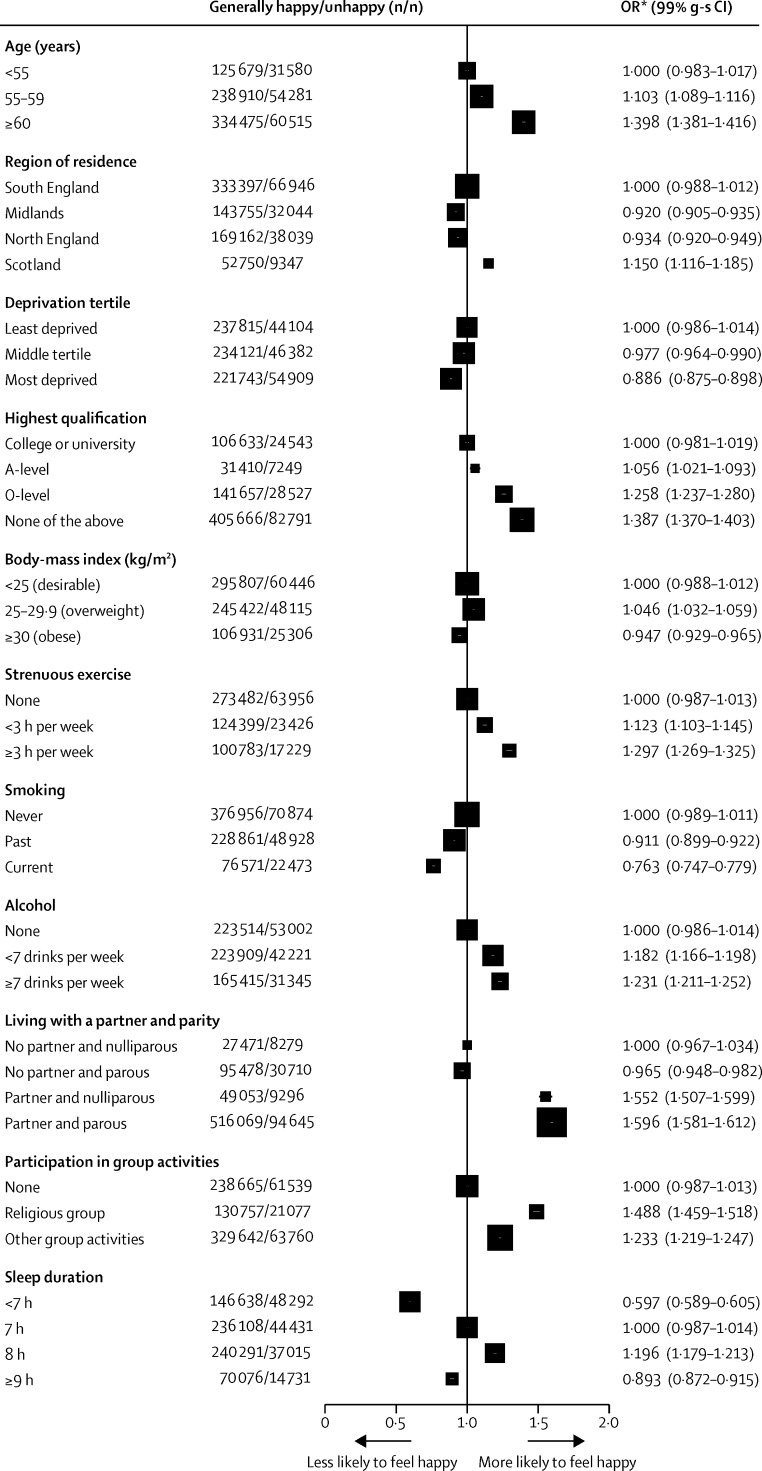
Correlates of being generally happy—relevance of personal and lifestyle characteristics at baseline Analysis for whole population (N=845 440), including women later excluded for life-threatening health disorders. *ORs are adjusted for age, region, area deprivation, body-mass index, qualifications, strenuous exercise, smoking, alcohol, living with a partner, parity, participation in group activities, and sleep duration. OR=odds ratio. g-s CI=group-specific confidence interval.

**Figure 2:**
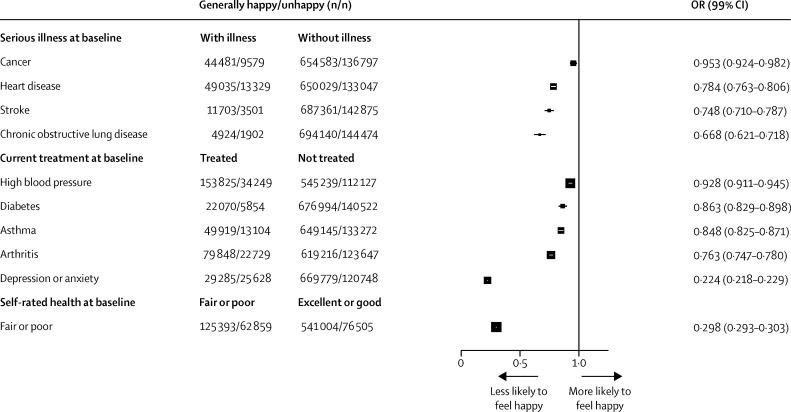
Correlates of being generally happy—relevance of various indices of health at baseline Analysis for whole population (N=845 440), including women later excluded for life-threatening health disorders. ORs are adjusted for age, region, area deprivation, body-mass index, qualifications, strenuous exercise, smoking, alcohol, living with a partner, parity, participation in group activities, and sleep duration. OR=odds ratio.

The strongest sociodemographic and lifestyle correlates of being generally happy were increasing age, having fewer educational qualifications, doing strenuous exercise, not smoking, living with a partner, and participating in religious and other group activities (figure 1). The relation between happiness and the number of hours of sleep was J-shaped, with women reporting about 8 h sleep most likely to be generally happy. Each of the indices of ill health at baseline was associated with unhappiness (figure 2). Of all factors shown in figure 1 or figure 2, the strongest associations with reported unhappiness were treatment for depression or anxiety and reporting only fair or poor general health (figure 2).

Women were followed for a mean of 9·6 years (SD 1·9) after completing the questionnaire about happiness. Including women with and without illness at baseline, 48 314 deaths were recorded in this time. Compared with those reporting being happy most of the time, women who had reported being unhappy had excess all-cause mortality when adjusted only for age (RR 1·36, 95% CI 1·33–1·40). Simultaneous adjustment for the sociodemographic and lifestyle factors in figure 1 and the indices of health in figure 2 completely eliminated this excess (fully adjusted RR 0·95, 0·93–0·98; appendix p 4). However, these analyses include women who already had life-threatening diseases at baseline. Hence, our subsequent analyses exclude the 125 769 women who at baseline already had heart disease, stroke, cancer, or chronic obstructive airways disease. These excluded women had three times the death rate of the women without any such illnesses (age-adjusted RR 2·91, 95% CI 2·85–2·96), and are omitted from the main analyses below.

Of the remaining 719 671 women (median age 59 years, IQR 55–63), 4% (31 531) died during follow-up. In response to the question at baseline about how often they felt happy, 39% (282 619) reported being happy most of the time, 44% (315 874) usually happy, and 17% (121 178) unhappy. In crude analyses adjusted only for age, unhappiness remained associated with increased mortality (RR 1·29, 95% CI 1·25–1·33; table). This excess risk was partly accounted for by associations with various personal characteristics (appendix p 5). Self-rated health was, however, the key characteristic. Poor health at baseline was strongly associated with unhappiness at baseline (figure 2) and once we adjusted for self-rated health, unhappiness was no longer significantly associated with all-cause mortality (RR 1·02, 0·98–1·05; table). After simultaneous adjustment for all sociodemographic and lifestyle factors in figure 1 (personal characteristics) and all indices of health in figure 2, the association vanished (fully adjusted RR 0·98, 0·94–1·01, for all-cause mortality [table]; 0·97, 0·87–1·10, for ischaemic heart disease mortality; and 0·98, 0·93–1·02, for cancer mortality [appendix pp 8–9]).

Further details of this multivariate adjustment are available (appendix p 5), showing that after adjusting for age, additional adjustment for each single personal characteristic in figure 1 changed the RR estimate only slightly (adjustment for smoking had the greatest effect). Simultaneous adjustment for all personal characteristics, however, halved the association between unhappiness and mortality (RR 1·14, 95% CI 1·11–1·18). Adjustment just for being treated for common health disorders (hypertension, diabetes, asthma, arthritis, depression, or anxiety), particularly depression or anxiety, also weakened the relationship (RR 1·21, 1·17–1·25; appendix p 5). The main findings were essentially unchanged in sensitivity analysis that ignored the first 5 years of follow-up (appendix p 6).

**Figure 3:**
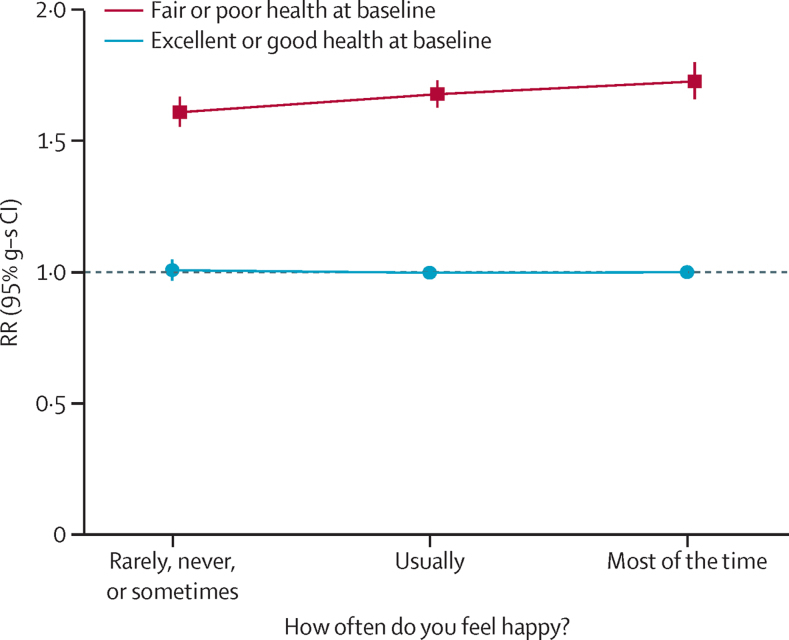
RR of all-cause mortality by self-rated health and happiness Includes 719 671 women (31 531 deaths). Excludes women with cancer, heart disease, stroke, or chronic obstructive airways disease at baseline. RRs are adjusted for age, region, area deprivation, body-mass index, qualifications, strenuous exercise, smoking, alcohol, living with a partner, parity, participation in group activities, and sleep duration. Women who reported being in good or excellent health and happy most of the time are the reference group (RR=1·0). RR=rate ratio. g-s CI=group-specific confidence interval.

**Figure 4:**
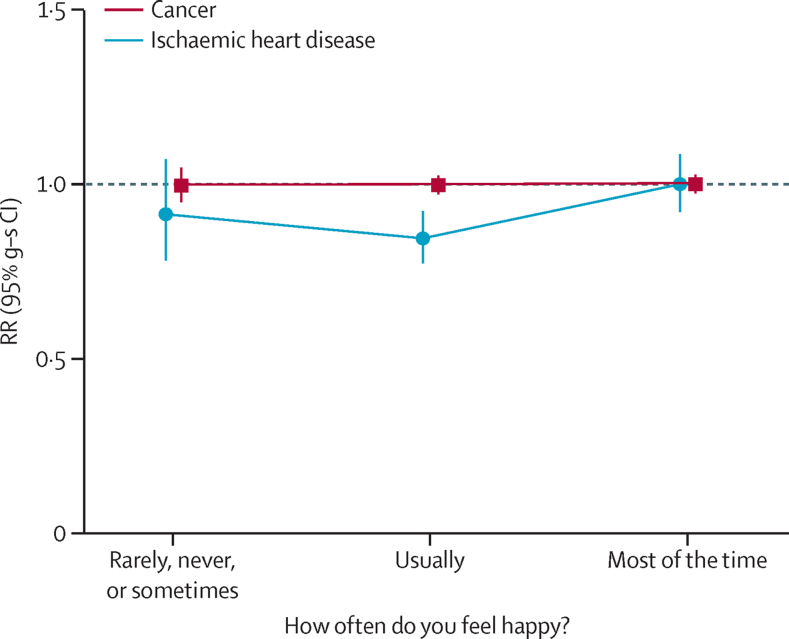
Risk of ischaemic heart disease mortality and cancer mortality by happiness in women who rated their health as good or excellent at baseline Includes 550 737 women (1253 ischaemic heart disease deaths, 12 943 cancer deaths). Excludes women with cancer, heart disease, stroke, or chronic obstructive airways disease at baseline, and women who rated their health as poor or fair at baseline. RRs are adjusted for age, region, area deprivation, body-mass index, qualifications, strenuous exercise, smoking, alcohol, living with a partner, parity, participation in group activities, and sleep duration. Women who reported being happy most of the time are the reference group (RR=1·0). RR=rate ratio. g-s CI=group-specific confidence interval.

**Figure 5:**
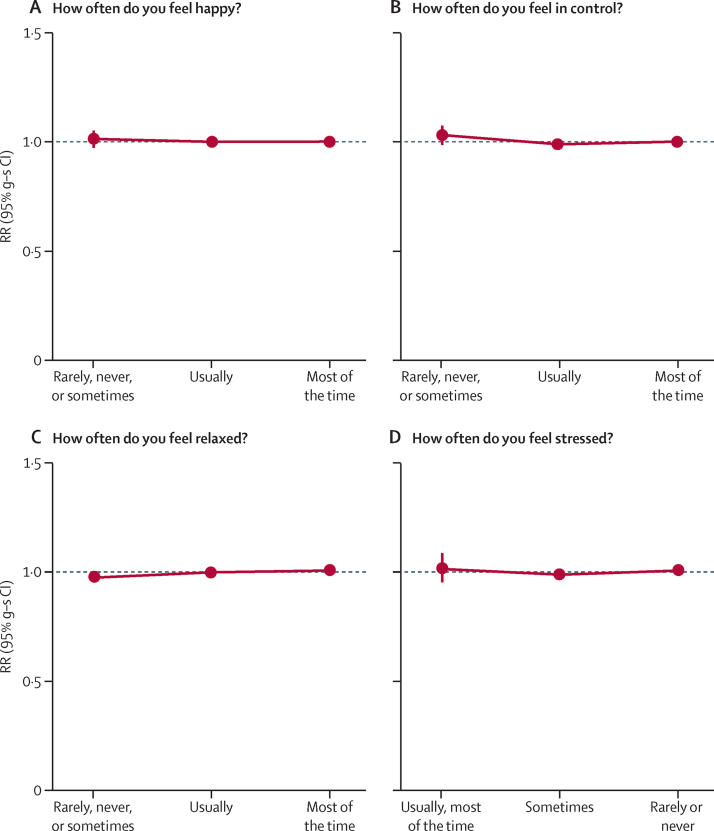
All-cause mortality by happiness and other measures of wellbeing in women who rated their health as good or excellent at baseline Includes 550 737 women (20 073 deaths). Excluding women with cancer, heart disease, stroke, or chronic obstructive airways disease at baseline and women who rated their health as poor or fair at baseline. RRs are adjusted for age, region, area deprivation, body-mass index, qualifications, strenuous exercise, smoking, alcohol, living with a partner, parity, participation in group activities, and sleep duration. The referenced groups (RR=1·0) were women who reported being happy most of the time (A); in control most of the time (B); relaxed most of the time (C); and rarely or never stressed (D). g-s CIs that are not visible are smaller than the solid circle. RR=rate ratio. g-s CI=group-specific confidence interval.

Because self-rated health was so strongly associated with both happiness and mortality, we examined the associations between happiness and mortality in women separately by self-rated health (table, figure 3, appendix pp 7–9, pp 13–15). All-cause mortality was substantially greater for the 20% (134 727 of 685 464) of women who reported that their health was fair or poor than for the remaining 80% (550 737) of women who reported good or excellent health (RR 1·67, 95% CI 1·63–1·71). Within each category of self-rated health there was no significant excess mortality in individuals who reported being unhappy (table, figure 3). For women who reported only fair or poor health, mortality was actually lower in those who reported being unhappy compared with those who were happy most of the time, but these findings might be biased by some unhappy women tending to rate their general health worse than it was, thus producing a spuriously low mortality associated with being unhappy. Women reporting being in good or excellent health are less liable to such a potential bias, and we give results for these women only in figure 4 and figure 5, with results for women reporting fair or poor health in the appendix.

Among 550 737 women reporting good or excellent health, 1253 died from ischaemic heart disease and 12 943 from cancer; among these women, unhappiness was not associated with mortality from either cause (figure 4). Nor was unhappiness associated with mortality from these causes for women who reported being in poor or fair health (appendix pp 8–9, pp 14–15).

Being treated for depression or anxiety was also strongly associated with self-reported unhappiness, so we did analyses separately for women who were and were not being treated for depression or anxiety at baseline (appendix pp 16–17). Again, unhappiness did not seem to be related to mortality in any of the subgroups of self-rated health.

We examined the associations of mortality with three other related subjective measures of wellbeing (being in control, relaxed, or not stressed). Correlates of each measure with sociodemographic, lifestyle, and health indices at baseline were similar to those found for happiness (appendix pp 18–19), even though the different measures were themselves not strongly correlated (appendix p 10). Among women reporting good or excellent health, not being in control, not being relaxed, and being stressed were all not associated with increased mortality (figure 5; appendix pp 11–12).

## Discussion

Poor health can cause unhappiness and poor health increases mortality, so unhappiness is associated with increased mortality. Additionally, unhappiness might correlate with some adverse lifestyle choices. After allowance for these associations our large prospective study shows no robust evidence that happiness itself reduces cardiac, cancer, or overall mortality.

There is no perfect or generally agreed way to measure happiness or related subjective indices of wellbeing. Different approaches thus limit comparability between studies. We used a single question about happiness with a four-point scale, whereas other investigators have used different measures.^10^, ^11^, ^12^, ^13^, ^14^ Nevertheless, we are able to show the validity of our measure in three ways. First, personal factors found to be associated with happiness in this study (figure 1, figure 2) were similar to those reported by others who used either single-item or multi-item measures of happiness—ie, women were more likely to report feeling happy if they were older,^15^, ^16^, ^17^ less deprived,^13^, ^15^, ^16^, ^18^ physically active,^11^, ^13^, ^19^, ^20^, ^21^ did not smoke,^3^, ^12^, ^20^, ^21^, ^22^ had a partner,^17^, ^18^, ^23^ belonged to a religious group or participated in social activities,^17^, ^18^ and had adequate sleep^3^, ^21^ (but not too much). Women were also less likely to be happy if they had poor self-rated health or were being treated for various common health disorders, particularly depression or anxiety.^16^, ^17^, ^18^, ^21^, ^24^ Second, in analyses that were adjusted only for age and not corrected for other factors, our measure of unhappiness was correlated with increased mortality. Third, the response to our single question on happiness was reasonably repeatable on resurvey 1 year later (weighted κ=0·62), a level of repeatability comparable with that reported by other researchers using both single-item and multiple-item measures.^3^, ^13^, ^25^ Also, there was minimal cross-over in responses 1 year apart between the two extremes of happy most of the time or unhappy.

Crude analyses, adjusting only for age, showed some excess mortality to be associated with unhappiness, but this excess was completely eliminated after additional adjustment for personal characteristics and for poor health at baseline (table). This was true for all-cause mortality and, separately, for mortality from ischaemic heart disease and from cancer. Far fewer women died from heart disease than from cancer and confounding was greater, so our null findings are less definite for heart disease than for cancer.

By far the most important adjustment factor was self-rated health. A systematic review of previous studies has confirmed that self-rated health predicts an increased risk of death, in agreement with our findings.^26^ Self-rated poor health was also strongly associated with unhappiness. Hence we examined the effects of happiness in categories of self-rated health, giving most weight to the findings in the many women who reported that they were in good or excellent health. In these women, unhappiness was not associated with an increased mortality. In women who reported that they were in fair or poor health, being unhappy was associated with a slightly lower mortality than being happy most of the time, but this finding could well be biased by unhappy women tending to rate their health as slightly worse than it actually was.

Unhappiness might cause some people to do things known to affect mortality adversely—eg, smoke or be inactive.^2^, ^3^ Hence, such variables might be mediators rather than confounders of the unhappiness–mortality association. Furthermore, some lifestyle factors such as inactivity and morbid obesity could cause unhappiness. However, adjustment for most of the behavioural factors, except smoking, resulted in little or no attenuation of the RR estimates for mortality, suggesting that even if these factors were mediating the association, their contribution is small. Adjustment for smoking caused a greater attenuation of the RR estimates than did adjustment for any other personal characteristics, so it is possible that part of the association between unhappiness and mortality, particularly for cancer mortality, might be mediated by smoking.

Some, but not all, other prospective studies have reported that happiness or related subjective measures of wellbeing are associated with lower all-cause mortality (panel).^4^, ^13^, ^14^, ^19^, ^20^, ^22^, ^27^, ^28^, ^29^ However, few of those reports excluded people with life-threatening illnesses at baseline and adjusted for self-rated health (or related measures of ill health) at baseline. Self-rated health was the most important confounding factor in our analyses; where other investigators adjusted for self-rated health, any apparent excess mortality associated with unhappiness was attenuated or disappeared completely.^27^ If there is inadequate allowance for ill health at baseline, any associations between happiness and lower mortality are likely to be artefactual.

Participants in the Million Women Study were slightly less likely to be from deprived areas than were the general UK population.^5^ However, at recruitment the cohort included about one in four women in England and Scotland in the eligible age range, indicating that findings should be generally applicable to middle-aged women in the UK. We provide no data about men or women of other ages.

It has been suggested that related subjective measures of wellbeing, including being in control, not being unduly stressed, or having positive or negative attitudes to life, could independently affect mortality.^4^, ^12^, ^14^, ^30^ However, just as for happiness, these associations were wholly accounted for by personal characteristics and ill health at baseline—after adjusting for these factors, any association with mortality was eliminated. We conclude that happiness and unhappiness have no material direct effect upon mortality.

## Figures and Tables

**Table:** Effects of adjustment for personal characteristics and various indices of health on the association between all-cause mortality and how often women reported being happy

		Unhappy*	**Usually happy**	**Happy most of the time**
**All women**				
Number of women		121 178	315 874	282 619
Number of deaths		6052	13 720	11 759
RR (95% CI) of all-cause mortality, adjusted for:				
	Age only	1·29 (1·25–1·33)	1·05 (1·03–1·08)	Ref
	Age and personal characteristics†	1·14 (1·11–1·18)	1·04 (1·02–1·07)	Ref
	Age and self-rated health‡	1·02 (0·98–1·05)	0·97 (0·95–1·00)	Ref
	Age, characteristics†, and self-rated health‡	0·97 (0·94–1·00)	0·98 (0·96–1·01)	Ref
	Age, characteristics†, self-rated health‡, and treatment for common health disorders§	0·98 (0·94–1·01)	0·99 (0·96–1·01)	Ref
**In women reporting poor or fair health at baseline**				
Number of women		46 547	56 447	31 733
Number of deaths		3193	4049	2364
RR (95% CI) of all-cause mortality, adjusted for:				
	Age only	0·99 (0·94–1·04)	0·97 (0·92–1·02)	Ref
	Age, characteristics†, and treatment for common health disorders§	0·93 (0·88–0·99)	0·97 (0·93–1·03)	Ref
**In women reporting good or excellent health at baseline**				
Number of women		68 762	244 488	237 487
Number of deaths		2509	8852	8712
RR (95% CI) of all-cause mortality, adjusted for:				
	Age only	1·06 (1·02–1·11)	1·00 (0·97–1·03)	Ref
	Age, characteristics†, and treatment for common health disorder§	1·01 (0·97–1·06)	1·00 (0·97–1·03)	Ref

Analyses are limited to the 719 671 women without cancer, heart disease, stroke, or chronic obstructive airways disease at baseline.

* Happy only sometimes, rarely, or never.

† Region of residence, area deprivation, educational qualifications, body-mass index, strenuous exercise, smoking, alcohol consumption, living with a partner, parity, participation in religious or other group activities, and sleep duration (appendix p 5 gives analyses adjusted for each of these separately).

‡ Self-rated health at baseline, in three categories: poor or fair, good, and excellent (the numbers for all women include the few who did not answer the question on self-rated health at baseline).

§ Treatment at baseline for high blood pressure, diabetes, asthma, arthritis, depression or anxiety (appendix p 5 gives the corresponding result adjusted only for treatment for depression or anxiety). RR=rate ratio. Ref=reference group.

## Panel Research in context

**Systematic review**

In November, 2014, we searched MEDLINE and PubMed for relevant reports with the terms “happiness”, “positive affect”, “well-being”, and “mortality” with no language or date restrictions. At least three comprehensive reviews had been done in the previous 6 years on the relation between happiness or related measures and mortality. We therefore did not do a new systematic review. The reviews and individual studies have reported inconsistent findings. Some suggest that happiness or related subjective measures of wellbeing are independently associated with decreased mortality, and some suggest no effect after adjustment for potential confounding factors such as poor health.

**Interpretation**

Some previous reports have confused cause and effect. Our findings show that unhappiness is associated with poor health mainly because poor health causes unhappiness and partly because unhappiness is associated with lifestyle factors such as smoking. After adjustment for these factors, no robust evidence remains that unhappiness or stress increase mortality or that being happy, relaxed, or in control reduces mortality.
