# Supplementary material for: Does happiness itself directly affect mortality? The prospective UK Million Women Study
Source: Lancet. 2016 Feb 27;387(10021):874–81. doi: 10.1016/S0140-6736(15)01087-9 (PMC5075047; doi:10.1016/S0140-6736(15)01087-9)
Supplement: Supplementary appendix [file mmc1.pdf]

THE LANCET

Supplementary appendix

This appendix formed part of the original submission and has been peer reviewed. We post it as supplied by the authors.

Supplement to: Liu B, Floud S, Pirie K, Green J, Peto R, Beral V, for the Million Women Study Collaborators. Does happiness itself directly affect mortality? The prospective UK Million Women Study. *Lancet* 2015; published online Dec 9. http://dx.doi.org/10.1016/S0140-6736(15)01087-9.

First page of online appendix intentionally left blank, to be replaced by a Lancet cover sheet

Online Appendix contents list; click on a page number to jump to it

Page 4
Webtable 1: All-cause mortality – Effects of adjustment for personal characteristics and indices of health on association of mortality with how often women felt happy
As Table 1 in the main text, but analysing all 845,440 women, including those with cancer, heart disease, stroke or chronic obstructive airways disease at baseline
Page 5
Webtable 2: All-cause mortality – Effects of separate adjustment for personal characteristics and indices of health on association of mortality with how often women felt happy
Analyses are restricted to the 719,671 women who remained after excluding those with cancer, heart disease, stroke or chronic obstructive airways disease at baseline.
Page 6
Webtable 3: Effects of excluding the first 5 years of follow-up and of adjusting for various factors on the association of mortality with how often women felt happy
Analyses are restricted to the 705,806 still being followed 5 years after baseline out of 719,671 women without cancer, heart disease, stroke or chronic obstructive airways disease at baseline.
Page 7
Webtable 4: Number of women according to how often they felt happy and their self-rated health at baseline
Numbers are restricted to the 685,464 women who remained after excluding those with cancer, heart disease, stroke or chronic obstructive airways disease at baseline and those who did not report on self-rated health at baseline
Page 8
Webtable 5: Ischaemic heart disease mortality – Effects of adjustment for personal characteristics and indices of health on association with how often women felt happy
Analyses are restricted to the 719,671 women without cancer, heart disease, stroke or chronic obstructive airways disease at baseline; shown for all women and stratified by self-rated health
Page 9
Webtable 6: Cancer mortality – Effects of adjustment for personal characteristics and indices of health on association with how often women felt happy
Analyses are restricted to the 719,671 women without cancer, heart disease, stroke or chronic obstructive airways disease at baseline; shown for all women and stratified by self-rated health
Page 10
Webtable 7: Spearman correlation coefficients between subjective measures of well-being (happy, in control, relaxed, stressed)
N=845,440 women. Spearman correlation coefficients are calculated on the four possible responses to each question (rarely/never, sometimes, usually, most of the time).
Page 11
***Webtable 8:* Four subjective measures of well-being (unhappy, not in control, not relaxed, stressed) –associations with all-cause mortality**
Analyses are restricted to the 719,671 women without cancer, heart disease, stroke or chronic obstructive airways disease at baseline.
Page 12
***Webtable 9:* Four subjective measures of well-being (unhappy, not in control, not relaxed, stressed) –associations with all-cause mortality**
Analyses are restricted to the 719,671 women without cancer, heart disease, stroke or chronic obstructive airways disease at baseline; with a different reference group to Webtable 8.

Page 13
Webfigure 1: All-cause mortality
Relative risk by self-rated health and happiness at baseline
Page 14
Webfigure 2: Ischaemic heart disease mortality
Relative risk by self-rated health and happiness at baseline
Page 15
Webfigure 3: Cancer mortality
Relative risk by self-rated health and happiness at baseline
Page 16
Webfigure 4: All-cause mortality in women treated for depression
Relative risk by self-rated health and happiness at baseline
Page 17
Webfigure 5: All-cause mortality in women NOT treated for depression
Relative risk by self-rated health and happiness at baseline
Page 18
Webfigure 6: Correlates of four subjective measures of well-being (unhappy, not in control, not relaxed, stressed)
By personal and lifestyle characteristics at baseline
Page 19
Webfigure 7: Correlates of four subjective measures of well-being (unhappy, not in control, not relaxed, stressed)
By various indices of health at baseline

***Webtable 1:* All-cause mortality – Effects of adjustment for personal characteristics and indices of health on association of mortality with how often women felt happy**

As Table 1 in the main text, but analysing all 845,440 women, including those with cancer, heart disease, stroke or chronic obstructive airways disease at baseline

	**Unhappy*** (N=146,376; 9,947 deaths)	**Usually happy** (N=369,738; 20,730 deaths)	**Happy most of the time** (N=329,326; 17,637 deaths)
**All-cause mortality (48,314 deaths); Relative risk (95% CI), adjusted for:**			
- Age only	1.36 (1.33-1.40)	1.06 (1.03-1.08)	1.00 (-)
- Age, personal characteristics†	1.20 (1.17-1.23)	1.05 (1.03-1.07)	1.00 (-)
- Age, self-rated health‡	0.99 (0.97-1.02)	0.95 (0.93-0.97)	1.00 (-)
- Age, characteristics,† health‡	0.95 (0.92-0.97)	0.96 (0.94-0.98)	1.00 (-)
**- Age, characteristics,† health,‡ treatment for common conditions§**	**0.95 (0.93-0.98)**	**0.97 (0.95-0.99)**	**1.00 (-)**

* Happy only sometimes, rarely or never

† Region of residence, deprivation, educational qualifications, body mass index, strenuous exercise, smoking, alcohol consumption, living with a partner, parity, participation in religious or other group activities, and sleep duration

‡ Self-rated health at baseline, in three categories: poor/fair, good and excellent

§ Treatment at baseline for high blood pressure, diabetes, asthma, arthritis, depression or anxiety

***Webtable 2:* All-cause mortality – Effects of separate adjustment for personal characteristics and indices of health on association of mortality with how often women felt happy**

Analyses are restricted to the 719,671 women who remained after excluding those with cancer, heart disease, stroke or chronic obstructive airways disease at baseline.

	**Unhappy*** (N=121,178; 6,052 deaths)	**Usually happy** (N=315,874; 13,720 deaths)	**Happy most of the time** (N=282,619; 11,759 deaths)
**All-cause mortality (31,531 deaths); Relative risk (95% CI), adjusted for:**			
- Age only	1.29 (1.25-1.33)	1.05 (1.03-1.08)	1.00 (-)
**- Age, various personal characteristics†**			
- Age, Region of residence	1.29 (1.25-1.33)	1.05 (1.03-1.08)	1.00 (-)
- Age, Deprivation quintile	1.26 (1.22-1.30)	1.06 (1.03-1.08)	1.00 (-)
- Age, Educational qualifications	1.30 (1.26-1.34)	1.06 (1.04-1.09)	1.00 (-)
- Age, Body mass index	1.28 (1.24-1.32)	1.06 (1.03-1.08)	1.00 (-)
- Age, Strenuous exercise	1.27 (1.23-1.31)	1.05 (1.03-1.08)	1.00 (-)
- Age, Smoking	1.20 (1.16-1.23)	1.03 (1.01-1.06)	1.00 (-)
- Age, Alcohol consumption	1.28 (1.24-1.32)	1.06 (1.03-1.09)	1.00 (-)
- Age, Living with a partner & parity	1.25 (1.21-1.29)	1.05 (1.02-1.07)	1.00 (-)
- Age, Participation in group activities	1.26 (1.22-1.30)	1.05 (1.02-1.08)	1.00 (-)
- Age, Sleep duration	1.29 (1.25-1.33)	1.06 (1.03-1.09)	1.00 (-)
- Age, and all the above characteristics	1.14 (1.11-1.18)	1.04 (1.02-1.07)	1.00 (-)
**- Age, various indices of health‡**			
- Age, Treatment for depression or anxiety	1.23 (1.20-1.27)	1.05 (1.02-1.07)	1.00 (-)
- Age, Treatment for high blood pressure, diabetes, asthma, arthritis, depression or anxiety	1.21 (1.17-1.25)	1.04 (1.02-1.07)	1.00 (-)
- Age, Self-rated health§	1.02 (0.98-1.05)	0.97 (0.95-1.00)	1.00 (-)

* Happy only sometimes, rarely or never

† Grouped as in Figure 1 in the main text ‡ Grouped as in Figure 2 in the main text

§ Self-rated health at baseline, in three categories: poor/fair, good and excellent

Webtable 3: **Effects of excluding the first 5 years of follow-up and of adjusting for various factors on the association of mortality with how often women felt happy**
Analyses are restricted to the 705,806 still being followed 5 years after baseline out of 719,671 women without cancer, heart disease, stroke or chronic obstructive airways disease at baseline.

	**Unhappy*** (N=118,575; 3,944 deaths)	**Usually happy** (N=309,863; 9,064 deaths)	**Happy most of the time** (N=277,368; 7,884 deaths)
**All-cause mortality (20,892 deaths); Relative risk (95% CI), adjusted for:**			
- Age only	1.26 (1.21-1.31)	1.04 (1.01-1.07)	1.00 (-)
- Age, personal characteristics†	1.12 (1.07-1.16)	1.03 (1.00-1.06)	1.00 (-)
- Age, self-rated health‡	1.01 (0.97-1.05)	0.96 (0.94-0.99)	1.00 (-)
- Age, characteristics,† health‡	0.96 (0.92-1.00)	0.98 (0.95-1.01)	1.00 (-)
**- Age, characteristics,† health,‡ treatment for common conditions§**	**0.97 (0.93-1.01)**	**0.98 (0.95-1.01)**	**1.00 (-)**

* Happy only sometimes, rarely or never

† Region of residence, deprivation, educational qualifications, body mass index, strenuous exercise, smoking, alcohol consumption, living with a partner, parity, participation in religious or other group activities, and sleep duration

‡ Self-rated health at baseline, in three categories: poor/fair, good and excellent

§ Treatment at baseline for high blood pressure, diabetes, asthma, arthritis, depression or anxiety

***Webtable 4:* Number of women according to how often they felt happy and their self-rated health at baseline**

Numbers are restricted to the 685,464 women who remained after excluding those with cancer, heart disease, stroke or chronic obstructive airways disease at baseline and those who did not report on self-rated health at baseline

	**Self-rated health**			
	Excellent	Good	Fair/Poor	**Total**
**How often do you feel happy?**				
Rarely/never/sometimes	7,825 *(6%)*	60,937 *(15%)*	46,547 *(35%)*	115,309 *(17%)*
Usually	45,695 *(35%)*	198,793 *(47%)*	56,447 *(42%)*	300,935 *(44%)*
Most of the time	77,415 *(59%)*	160,072 *(38%)*	31,733 *(24%)*	269,220 *(39%)*
**Total**	130,935 *(100%)*	419,802 *(100%)*	134,727 *(100%)*	685,464 *(100%)*

Column percentages are in brackets

Webtable 5: **Ischaemic heart disease mortality – Effects of adjustment for personal characteristics and indices of health on association with how often women felt happy**
Analyses are restricted to the 719,671 women without cancer, heart disease, stroke or chronic obstructive airways disease at baseline; shown for all women and stratified by self-rated health

	**Unhappy***	**Usually happy**	**Happy most of the time**
**Ischaemic heart disease (2292 deaths); Relative risk (95% CI), adjusted for:**	(N=121,178)	(N=315,874)	(N=282,619)
- Age only	1.44 (1.29-1.61)	0.93 (0.84-1.02)	1.00 (-)
- Age, personal characteristics†	1.17 (1.05-1.31)	0.92 (0.84-1.01)	1.00 (-)
- Age, self-rated health‡	0.98 (0.87-1.10)	0.81 (0.74-0.89)	1.00 (-)
- Age, characteristics,† health‡	0.91 (0.81-1.03)	0.84 (0.76-0.92)	1.00 (-)
**- Age, characteristics,† health,‡ treatment for common conditions§**	**0.97 (0.87-1.10)**	**0.85 (0.78-0.94)**	**1.00 (-)**
**In women reporting poor/fair health (876 deaths); Relative risk (95%CI), adjusted for:**	(N=46,547)	(N=56,447)	(N=31,733)
- Age only	1.03 (0.86-1.22)	0.89 (0.76-1.06)	1.00 (-)
- Age, characteristics†, treatment for common conditions§	1.02 (0.85-1.22)	0.93 (0.78-1.10)	1.00 (-)
**In women reporting good/excellent health (1253 deaths); Relative risk (95%CI), adjusted for:**	(N=68,762)	(N=244,488)	(N=237,487)
- Age only	1.01 (0.84-1.20)	0.84 (0.74-0.94)	1.00 (-)
- Age, characteristics†, treatment for common conditions§	0.94 (0.79-1.13)	0.85 (0.76-0.96)	1.00 (-)

* Happy only sometimes, rarely or never

† Region of residence, deprivation, educational qualifications, body mass index, strenuous exercise, smoking, alcohol consumption, living with a partner, parity, participation in religious or other group activities, and sleep duration

‡ Self-rated health at baseline, in three categories: poor/fair, good and excellent

§ Treatment at baseline for high blood pressure, diabetes, asthma, arthritis, depression or anxiety

***Webtable 6:* Cancer mortality – Effects of adjustment for personal characteristics and indices of health on association with how often women felt happy**

Analyses are restricted to the 719,671 women without cancer, heart disease, stroke or chronic obstructive airways disease at baseline; shown for all women and stratified by self-rated health

	**Unhappy***	**Usually happy**	**Happy most of the time**
**Cancer (18,414 deaths); Relative risk (95% CI), adjusted for:**	(N=121,178)	(N=315,874)	(N=282,619)
- Age only	1.13 (1.08-1.18)	1.03 (1.00-1.06)	1.00 (-)
- Age, personal characteristics†	1.03 (0.99-1.08)	1.02 (0.99-1.05)	1.00 (-)
- Age, self-rated health‡	1.00 (0.96-1.04)	0.99 (0.96-1.02)	1.00 (-)
- Age, characteristics,† health‡	0.97 (0.93-1.01)	1.00 (0.96-1.03)	1.00 (-)
**- Age, characteristics,† health,‡ treatment for common conditions§**	**0.98 (0.93-1.02)**	**1.00 (0.97-1.03)**	**1.00 (-)**
**In women reporting poor/fair health (4423 deaths); Relative risk (95%CI), adjusted for:**	(N=46,547)	(N=56,447)	(N=31,733)
- Age only	0.99 (0.91-1.07)	1.00 (0.93-1.08)	1.00 (-)
- Age, characteristics†, treatment for common conditions§	0.95 (0.87-1.03)	1.00 (0.93-1.08)	1.00 (-)
**In women reporting good/excellent health (12,943 deaths); Relative risk (95%CI), adjusted for:**	(N=68,762)	(N=244,488)	(N=237,487)
- Age only	1.04 (0.98-1.09)	1.00 (0.96-1.04)	1.00 (-)
- Age, characteristics†, treatment for common conditions§	1.00 (0.95-1.06)	1.00 (0.96-1.04)	1.00 (-)

* Happy only sometimes, rarely or never

† Region of residence, deprivation, educational qualifications, body mass index, strenuous exercise, smoking, alcohol consumption, living with a partner, parity, participation in religious or other group activities, and sleep duration

‡ Self-rated health at baseline, in three categories: poor/fair, good and excellent

§ Treatment at baseline for high blood pressure, diabetes, asthma, arthritis, depression or anxiety

***Webtable 7:* Spearman correlation coefficients between subjective measures of well-being (happy, in control, relaxed, stressed)**

N=845,440 women. Spearman correlation coefficients are calculated on the four possible responses to each question (rarely/never, sometimes, usually, most of the time).

**How often do you feel…**	Happy?	In control?	Relaxed?	Stressed?
Happy?	1.00			
In control?	0.49	1.00		
Relaxed?	0.60	0.54	1.00	
Stressed?	-0.34	-0.33	-0.43	1.00

***Webtable 8:* Four subjective measures of well-being (unhappy, not in control, not relaxed, stressed) – associations with all-cause mortality**

Analyses are restricted to the 719,671 women without cancer, heart disease, stroke or chronic obstructive airways disease at baseline.

	**Presence *versus* absence of 4 subjective measures of well-being**			
	**…unhappy*** (6,052 deaths)	**…not in control*** (5,223 deaths)	**…not relaxed*** (9,382 deaths)	**…stressed*** (2,892 deaths)
**All-cause mortality (31,531 deaths); Relative risk (95% CI), adjusted for:**				
- Age only	1.29 (1.25-1.33)	1.24 (1.20-1.29)	1.12 (1.09-1.16)	1.28 (1.22-1.34)
- Age, personal characteristics†	1.14 (1.11-1.18)	1.16 (1.12-1.20)	1.07 (1.04-1.10)	1.12 (1.07-1.17)
- Age, self-rated health‡	1.02 (0.98-1.05)	1.01 (0.97-1.04)	0.89 (0.86-0.92)	0.96 (0.91-1.00)
- Age, characteristics,† health‡	0.97 (0.94-1.00)	1.01 (0.97-1.04)	0.91 (0.88-0.94)	0.93 (0.88-0.97)
**- Age, characteristics,† health,‡ treatment for common conditions§**	**0.98 (0.94-1.01)**	**1.01 (0.98-1.05)**	**0.91 (0.88-0.94)**	**0.92 (0.88-0.97)**

* For the questions about how often women felt happy, in control, or relaxed, the relative risks compare the replies Sometimes/Rarely/Never *versus* Most of the time (Appendix Webtable9 gives relative risks for Sometimes/Rarely/Never *versus* Most of the time/Usually). For stress, the relative risks compare the replies Usually/Most of the time *versus* Rarely/Never.

† Region of residence, deprivation, educational qualifications, body mass index, strenuous exercise, smoking, alcohol consumption, living with a partner, parity, participation in religious or other group activities, and sleep duration

‡ Self-rated health at baseline, in three categories: poor/fair, good and excellent

§ Treatment at baseline for high blood pressure, diabetes, asthma, arthritis, depression or anxiety

Webtable 9: **Four subjective measures of well-being (unhappy, not in control, not relaxed, stressed) – associations with all-cause mortality**

Analyses are restricted to the 719,671 women without cancer, heart disease, stroke or chronic obstructive airways disease at baseline; with a different reference group to Webtable 8.

	**Presence *versus* absence of 4 subjective measures of well-being**			
	**…unhappy*** (6,052 deaths)	**…not in control*** (5,223 deaths)	**…not relaxed*** (9,382 deaths)	**…stressed*** (2,892 deaths)
**All-cause mortality (31,531 deaths); Relative risk (95% CI), adjusted for:**				
- Age only	1.26 (1.22-1.29)	1.26 (1.22-1.30)	1.13 (1.10-1.16)	1.28 (1.24-1.34)
- Age, personal characteristics†	1.11 (1.08-1.15)	1.15 (1.12-1.19)	1.06 (1.03-1.09)	1.12 (1.07-1.16)
- Age, self-rated health‡	1.03 (1.00-1.06)	1.05 (1.02-1.09)	0.94 (0.92-0.97)	1.04 (1.00-1.08)
- Age, characteristics,† health‡	0.98 (0.95-1.01)	1.03 (1.00-1.06)	0.93 (0.91-0.96)	0.98 (0.94-1.02)
**- Age, characteristics,† health,‡ treatment for common conditions§**	**0.98 (0.95-1.01)**	**1.03 (1.00-1.06)**	**0.93 (0.91-0.96)**	**0.97 (0.93-1.01)**

* For the questions about how often women felt happy, in control, or relaxed, the relative risks compare the replies Sometimes/Rarely/Never *versus* Usually/Most of the time. For stress, the relative risks compare the replies Usually/Most of the time *versus* Sometimes/Rarely/Never.

† Region of residence, deprivation, educational qualifications, body mass index, strenuous exercise, smoking, alcohol consumption, living with a partner, parity, participation in religious or other group activities, and sleep duration

‡ Self-rated health at baseline, in three categories: poor/fair, good and excellent

§ Treatment at baseline for high blood pressure, diabetes, asthma, arthritis, depression or anxiety

Webfigure 1: All-cause mortality: Relative risk* by self-rated health and happiness

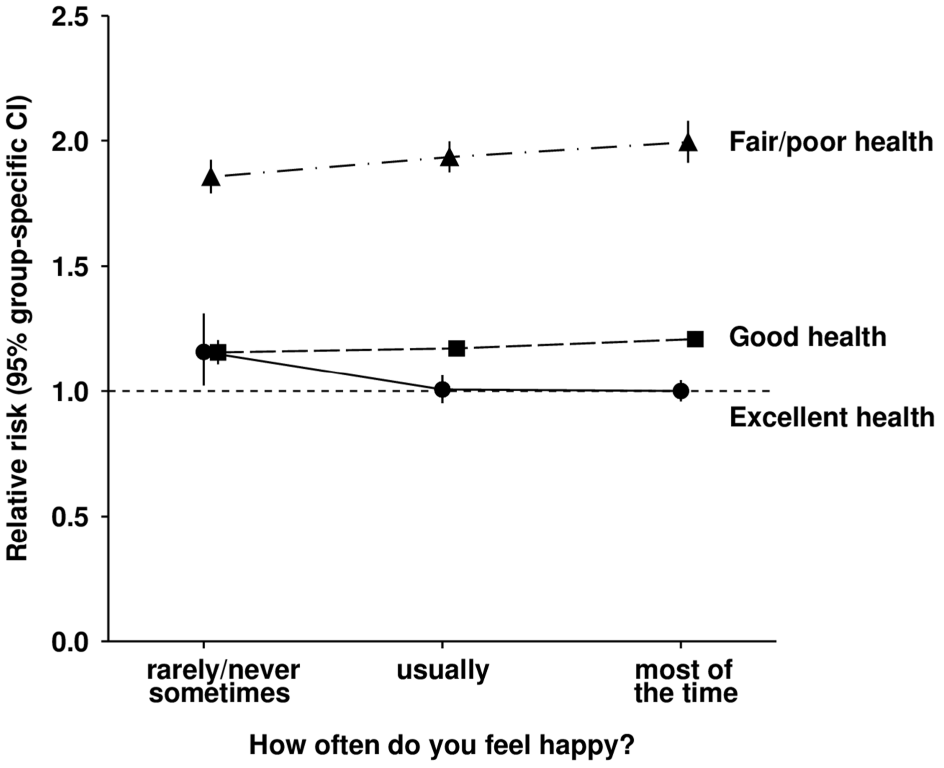

N=719,671 women (31,531 deaths). Excluding women with cancer, heart disease, stroke or chronic obstructive airways disease at baseline.

* Relative risk, adjusted for age, region, deprivation, body mass index, qualifications, strenuous exercise, smoking, alcohol, living with a partner, parity, participation in group activities and sleep duration; women who reported being in excellent health and happy most of the time are the reference group (relative risk=1.0).

Webfigure 2: Ischaemic heart disease mortality - Relative risk* by self-rated health and happiness

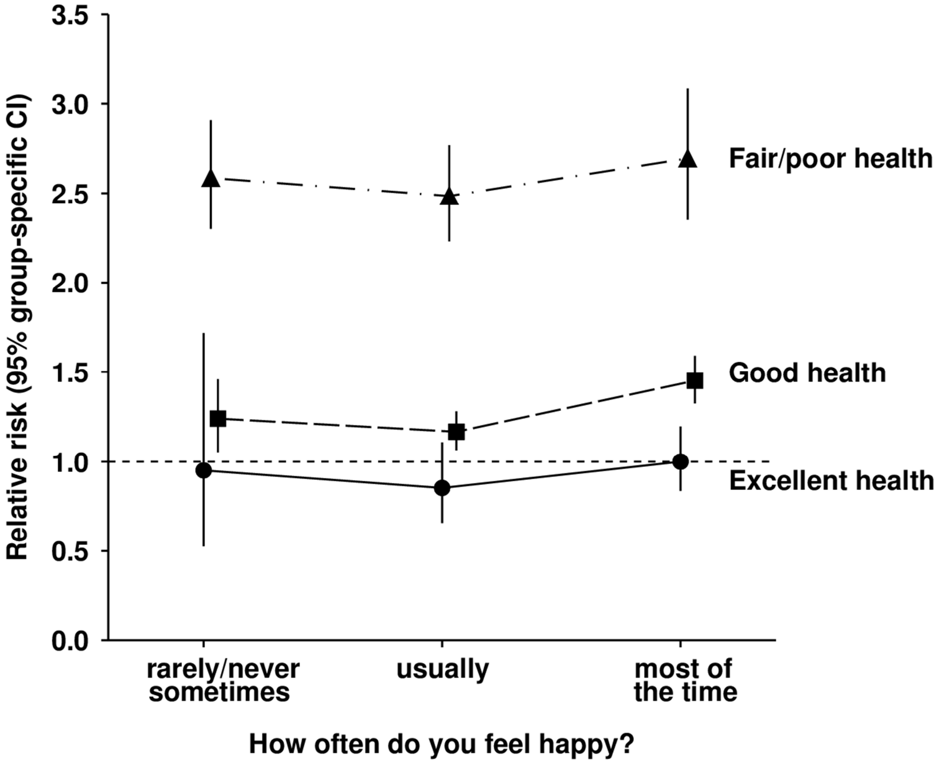

N=719,671 women (2292 deaths). Excluding women with cancer, heart disease, stroke or chronic obstructive airways disease at baseline.

* Relative risk, adjusted for age, region, deprivation, body mass index, qualifications, strenuous exercise, smoking, alcohol, living with a partner, parity, participation in group activities and sleep duration; women who reported being in excellent health and happy most of the time are the reference group (relative risk=1.0)

Webfigure 3: Cancer mortality - Relative risk* by self-rated health and happiness

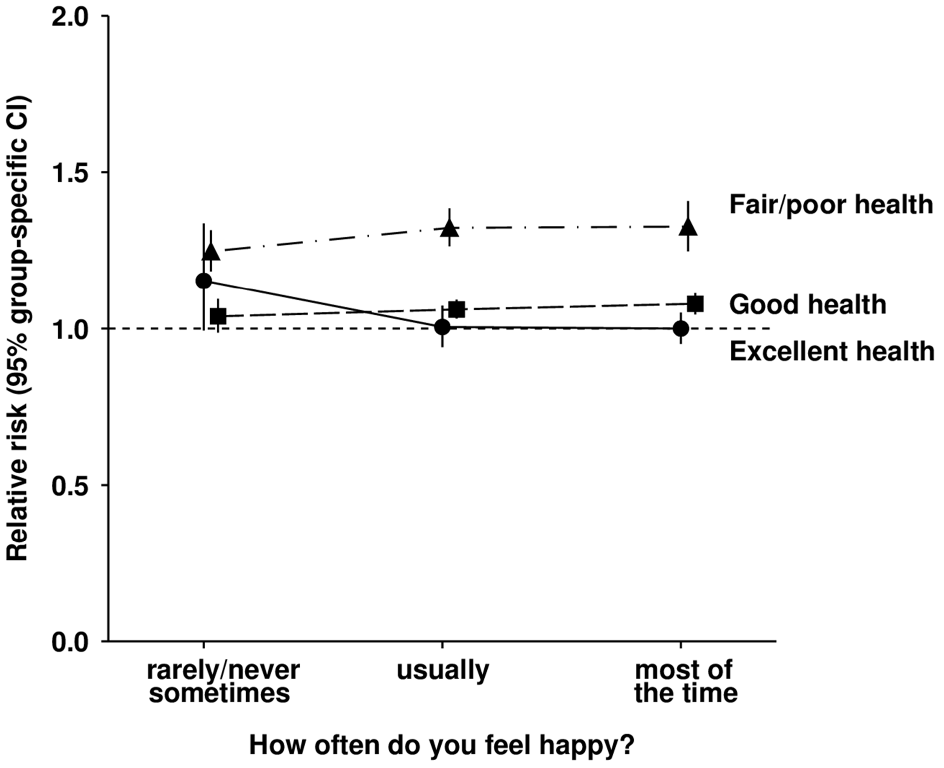

N=719,671 women (18,414 deaths). Excluding women with cancer, heart disease, stroke or chronic obstructive airways disease at baseline.

* Relative risk, adjusted for age, region, deprivation, body mass index, qualifications, strenuous exercise, smoking, alcohol, living with a partner, parity, participation in group activities and sleep duration; women who reported being in excellent health and happy most of the time are the reference group (relative risk=1.0)

Webfigure 4: All-cause mortality in women treated for depression - Relative risk* by self-rated health and happiness

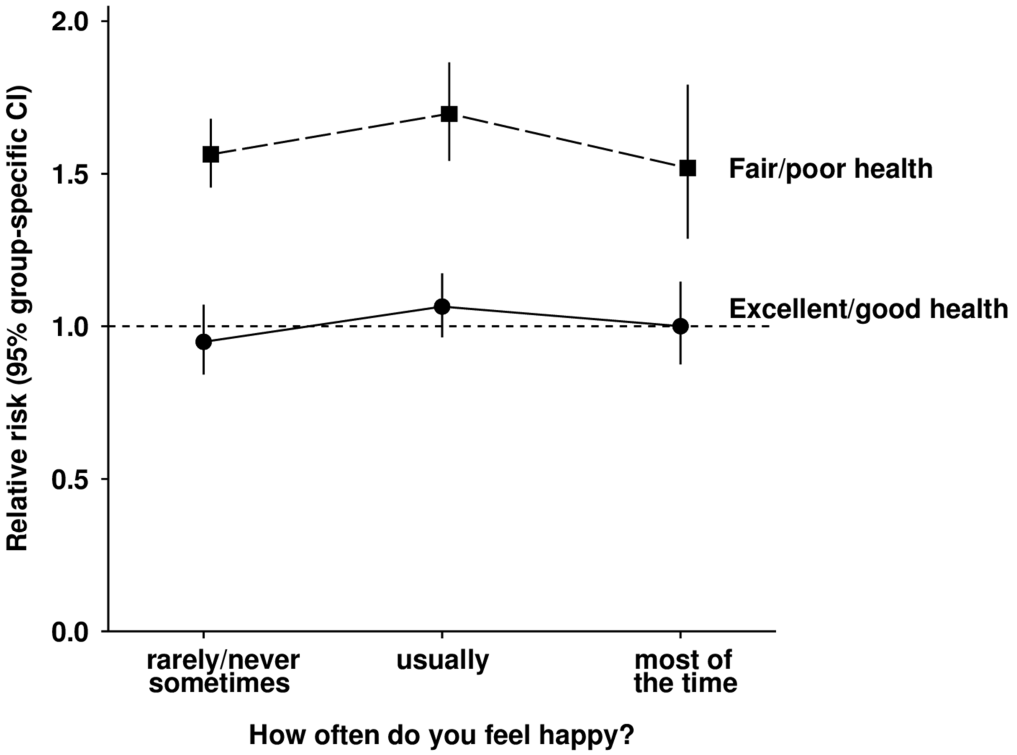

N=41,301 women (2332 deaths). Excluding women with cancer, heart disease, stroke or chronic obstructive airways disease, and women not treated for depression at baseline.

* Relative risk, adjusted for age, region, deprivation, body mass index, qualifications, strenuous exercise, smoking, alcohol, living with a partner, parity, participation in group activities and sleep duration; women who reported being in good or excellent health and happy most of the time are the reference group (relative risk=1.0)

Webfigure 5: All-cause mortality in women NOT treated for depression - Relative risk* by self-rated health and happiness

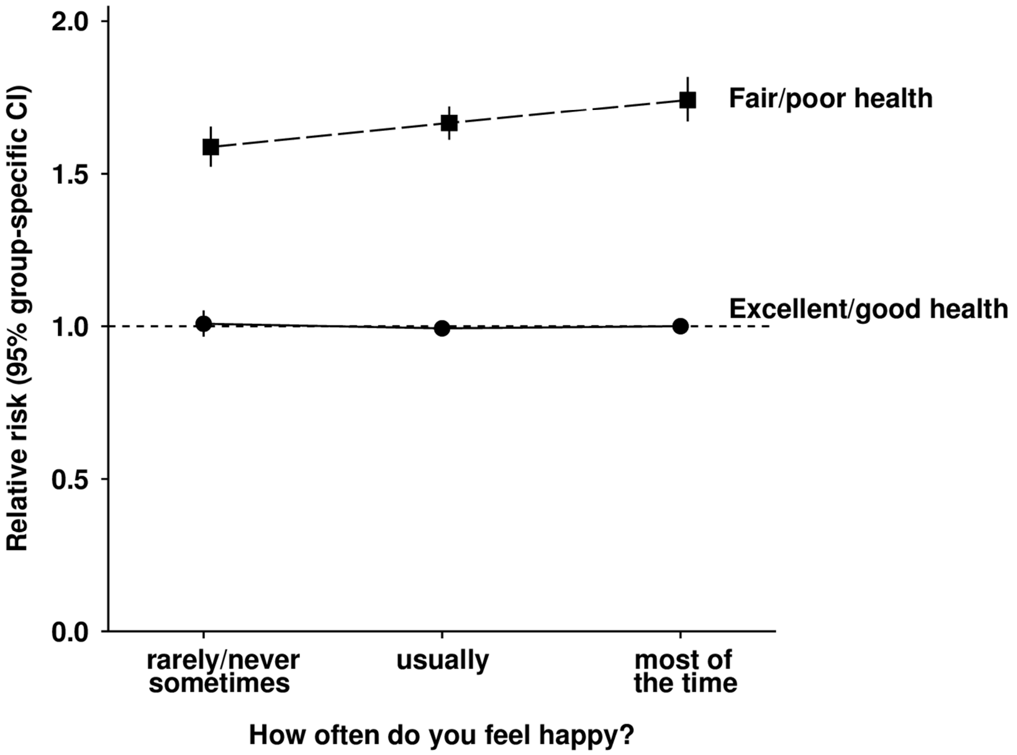

N=644,163 women (27,347 deaths). Excluding women with cancer, heart disease, stroke or chronic obstructive airways disease, and women treated for depression at baseline.

* Relative risk, adjusted for age, region, deprivation, body mass index, qualifications, strenuous exercise, smoking, alcohol, living with a partner, parity, participation in group activities and sleep duration; women who reported being in good or excellent health and happy most of the time are the reference group (relative risk=1.0)

Webfigure 6: Correlates of feeling generally happy, in control, relaxed and unstressed by personal and lifestyle characteristics at baseline.

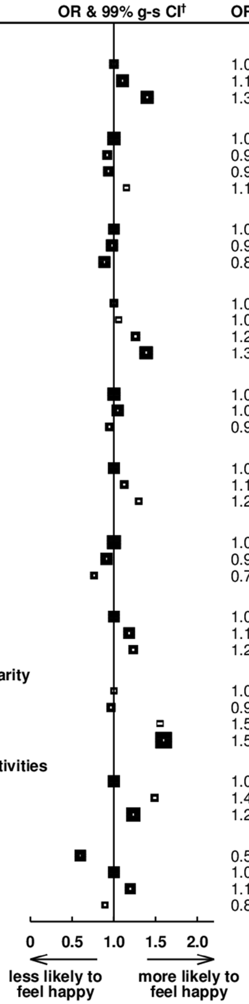

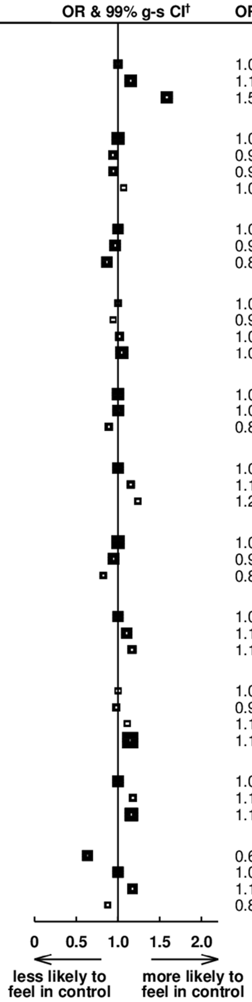

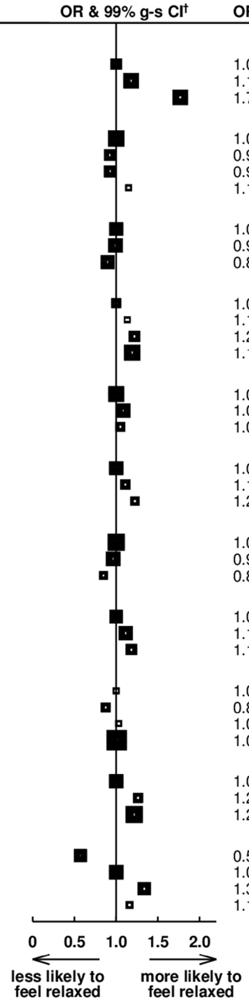

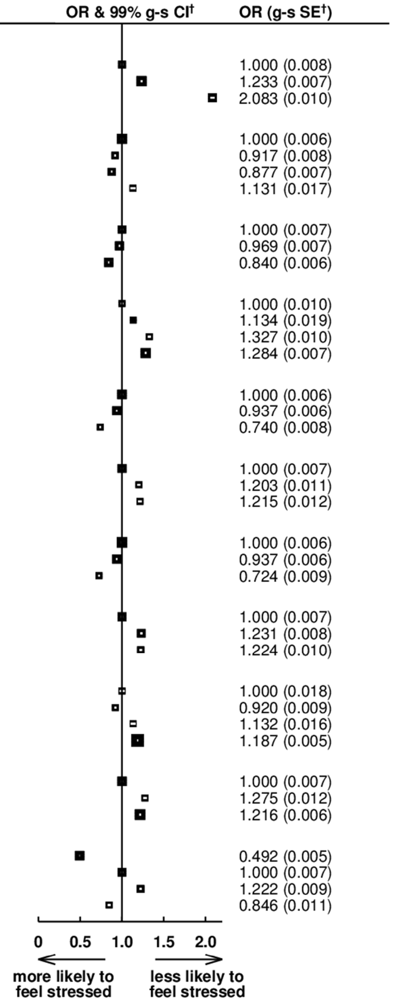

How often do you feel...	Happy? (usually/most of the time vs sometimes/rarely/never) OR (g-s SE†)	In control? (usually/most of the time vs sometimes/rarely/never) OR (g-s SE†)	Relaxed? (usually/most of the time vs sometimes/rarely/never) OR (g-s SE†)	Unstressed? (sometimes/rarely/never vs usually/most of the time) OR (g-s SE†)
**Age (years)**				
<55	1.000 (0.007)	1.000 (0.007)	1.000 (0.005)	1.000 (0.008)
55-59	1.103 (0.005)	1.152 (0.005)	1.180 (0.004)	1.233 (0.007)
60+	1.398 (0.006)	1.585 (0.006)	1.768 (0.005)	2.083 (0.010)
**Region of residence**				
South England	1.000 (0.005)	1.000 (0.005)	1.000 (0.004)	1.000 (0.006)
Midlands	0.920 (0.006)	0.938 (0.006)	0.923 (0.005)	0.917 (0.008)
North England	0.934 (0.006)	0.944 (0.006)	0.928 (0.005)	0.877 (0.007)
Scotland	1.150 (0.012)	1.067 (0.012)	1.149 (0.010)	1.131 (0.017)
**Deprivation tertile**				
Least deprived	1.000 (0.005)	1.000 (0.006)	1.000 (0.004)	1.000 (0.007)
Middle tertile	0.977 (0.005)	0.966 (0.005)	0.991 (0.004)	0.969 (0.007)
Most deprived	0.886 (0.005)	0.866 (0.005)	0.898 (0.004)	0.840 (0.006)
**Highest qualification**				
College/university	1.000 (0.007)	1.000 (0.008)	1.000 (0.006)	1.000 (0.010)
A level	1.056 (0.014)	0.941 (0.014)	1.133 (0.012)	1.134 (0.019)
O level	1.258 (0.007)	1.018 (0.007)	1.218 (0.006)	1.327 (0.010)
None of the above	1.387 (0.005)	1.044 (0.005)	1.192 (0.004)	1.284 (0.007)
**Body mass index (kg/m^2)**				
<25 (desirable)	1.000 (0.005)	1.000 (0.005)	1.000 (0.004)	1.000 (0.006)
25-29.9 (overweight)	1.046 (0.005)	1.003 (0.005)	1.083 (0.004)	0.937 (0.006)
30+ (obese)	0.947 (0.007)	0.889 (0.007)	1.050 (0.006)	0.740 (0.008)
**Strenuous exercise**				
None	1.000 (0.005)	1.000 (0.005)	1.000 (0.004)	1.000 (0.007)
<3 hours/week	1.123 (0.008)	1.153 (0.008)	1.109 (0.006)	1.203 (0.011)
3+ hours/week	1.297 (0.010)	1.238 (0.009)	1.225 (0.007)	1.215 (0.012)
**Smoking**				
Never	1.000 (0.004)	1.000 (0.005)	1.000 (0.004)	1.000 (0.006)
Past	0.911 (0.005)	0.947 (0.005)	0.963 (0.004)	0.937 (0.006)
Current	0.763 (0.007)	0.823 (0.008)	0.848 (0.007)	0.724 (0.009)
**Alcohol**				
None	1.000 (0.005)	1.000 (0.006)	1.000 (0.005)	1.000 (0.007)
<7 drinks/week	1.182 (0.006)	1.103 (0.006)	1.114 (0.005)	1.231 (0.008)
7+ drinks/week	1.231 (0.007)	1.167 (0.007)	1.182 (0.006)	1.224 (0.010)
**Living with a partner & parity**				
No partner & nulliparous	1.000 (0.013)	1.000 (0.014)	1.000 (0.012)	1.000 (0.018)
No partner & parous	0.965 (0.007)	0.979 (0.008)	0.874 (0.006)	0.920 (0.009)
Partner & nulliparous	1.552 (0.014)	1.110 (0.012)	1.031 (0.010)	1.132 (0.016)
Partner & parous	1.596 (0.005)	1.145 (0.004)	1.006 (0.003)	1.187 (0.005)
**Participation in group activities**				
None	1.000 (0.005)	1.000 (0.005)	1.000 (0.004)	1.000 (0.007)
Religious group	1.488 (0.009)	1.178 (0.008)	1.261 (0.007)	1.275 (0.012)
Other group activities	1.233 (0.005)	1.160 (0.005)	1.217 (0.004)	1.216 (0.006)
**Sleep duration**				
<7 hours	0.597 (0.004)	0.635 (0.005)	0.571 (0.004)	0.492 (0.005)
7 hours	1.000 (0.005)	1.000 (0.005)	1.000 (0.004)	1.000 (0.007)
8 hours	1.196 (0.006)	1.173 (0.006)	1.337 (0.005)	1.222 (0.009)
9+ hours	0.893 (0.009)	0.876 (0.009)	1.160 (0.009)	0.846 (0.011)

* Adjusted, where appropriate, for age, region, deprivation, body mass index, qualifications, strenuous exercise, smoking, alcohol, living with a partner, parity, participation in group activities and sleep duration
† g-s CI: group-specific confidence interval; g-s SE: group-specific standard error

Webfigure 7: Correlates of feeling generally happy, in control, relaxed and unstressed by various indices of health at baseline.

How often do you feel...	Happy? (usually/most of the time vs sometimes/rarely/never) OR (SE)	In control? (usually/most of the time vs sometimes/rarely/never) OR (SE)	Relaxed? (usually/most of the time vs sometimes/rarely/never) OR (SE)	Unstressed? (sometimes/rarely/never vs usually/most of the time) OR (SE)
**Serious illness at baseline**				
cancer	0.953 (0.012)	0.931 (0.012)	0.981 (0.010)	0.947 (0.016)
heart disease	0.784 (0.009)	0.777 (0.010)	0.744 (0.008)	0.671 (0.011)
stroke	0.748 (0.017)	0.667 (0.017)	0.766 (0.016)	0.659 (0.021)
chronic obstructive airways disease	0.668 (0.023)	0.676 (0.025)	0.668 (0.021)	0.599 (0.027)
**Current treatment at baseline**				
high blood pressure	0.928 (0.007)	0.908 (0.007)	0.836 (0.006)	0.819 (0.009)
diabetes	0.863 (0.015)	0.865 (0.015)	0.925 (0.013)	0.831 (0.018)
asthma	0.848 (0.010)	0.851 (0.010)	0.805 (0.008)	0.731 (0.011)
arthritis	0.763 (0.007)	0.821 (0.008)	0.751 (0.006)	0.738 (0.009)
depression/anxiety	0.224 (0.005)	0.244 (0.005)	0.206 (0.005)	0.181 (0.005)
**Self-rated health at baseline**				
fair/poor	0.298 (0.004)	0.340 (0.004)	0.321 (0.004)	0.298 (0.005)

OR & 99% CI (axis 0, 0.5, 1.0)

Happy: ← less likely to feel happy | more likely to feel happy →

In control: ← less likely to feel in control | more likely to feel in control →

Relaxed: ← less likely to feel relaxed | more likely to feel relaxed →

Unstressed: ← more likely to feel stressed | less likely to feel stressed →

* Adjusted, where appropriate, for age, region, deprivation, body mass index, qualifications, strenuous exercise, smoking, alcohol, living with a partner, parity, participation in group activities and sleep duration
